# A mobile clinic approach to the delivery of community-based mental health services in rural Haiti

**DOI:** 10.1371/journal.pone.0199313

**Published:** 2018-06-20

**Authors:** J. Reginald Fils-Aimé, David J. Grelotti, Tatiana Thérosmé, Bonnie N. Kaiser, Giuseppe Raviola, Yoldie Alcindor, Jennifer Severe, Emmeline Affricot, Katherine Boyd, Rupinder Legha, Shin Daimyo, Stephanie Engel, Eddy Eustache

**Affiliations:** 1 Zanmi Lasante, Mirebalais, Haiti; 2 Department of Psychiatry, University of California San Diego, La Jolla, CA, United States of America; 3 Duke Global Health Institute, Duke University, Durham, NC, United States of America; 4 Partners In Health, Boston, MA, United States of America; 5 Department of Global Health and Social Medicine, Harvard Medical School, Boston, MA, United States of America; 6 Department of Psychiatry, Columbia University, New York, NY, United States of America; 7 Colorado School of Public Health, Denver, CO, United States of America; 8 Department of Psychiatry, University of California Los Angeles, Los Angeles, CA, United States of America; 9 Yale School of Nursing, New Haven, CT, United States of America; 10 Private practice, Cambridge, MA, United States of America; University of Toronto, CANADA

## Abstract

This study evaluates the use of a mental health mobile clinic to overcome two major challenges to the provision of mental healthcare in resource-limited settings: the shortage of trained specialists; and the need to improve access to safe, effective, and culturally sound care in community settings. Employing task-shifting and supervision, mental healthcare was largely delivered by trained, non-specialist health workers instead of specialists. A retrospective chart review of 318 unduplicated patients assessed and treated during the mobile clinic’s first two years (January 2012 to November 2013) was conducted to explore outcomes. These data were supplemented by a quality improvement questionnaire, illustrative case reports, and a qualitative interview with the mobile clinic’s lead community health worker. The team evaluated an average of 42 patients per clinic session. The most common mental, neurological, or substance abuse (MNS) disorders were depression and epilepsy. Higher follow-up rates were seen among those with diagnoses of bipolar disorder and neurological conditions, while those with depression or anxiety had lower follow-up rates. Persons with mood disorders who were evaluated on at least two separate occasions using a locally developed depression screening tool experienced a significant reduction in depressive symptoms. The mental health mobile clinic successfully treated a wide range of MNS disorders in rural Haiti and provided care to individuals who previously had no consistent access to mental healthcare. Efforts to address these common barriers to the provision of mental healthcare in resource-limited settings should consider supplementing clinic-based with mobile services.

## Introduction

The burden of mental, neurological, and substance use (MNS) disorders often exceeds the capacity of healthcare systems, creating a “treatment gap”[1]. The treatment gap for MNS disorders is greatest in low- and middle-income countries (LMICs), where three-fourths of people with serious mental illness and epilepsy cannot access basic treatment services [2,3]. This is especially worrisome because MNS disorders are among the most disabling conditions worldwide [4]. Barriers to closing this treatment gap in LMICs are the vast scarcity of skilled human resources, large inequities and inefficiencies in resource distribution, and stigma associated with psychiatric illness, including among providers [5–7].

Task-shifting, or the transfer of knowledge, skills, and tasks from specialist to non-specialist health workers, can expand treatment to underserved areas [8]. Task-shifting has been successfully used for depression, post-traumatic stress disorder, alcohol-use disorders, and dementia [9–12].

Mobile clinics deliver care to hard-to-reach or disaster-affected populations [13–15]. They are flexible: personnel and services can be tailored to geographic location, culture, language, gender and/or choice of services to promote patient-centered care [16]. While some mobile clinics offer supplementary services for persons with MNS disorder [13–15,17], we found nothing in the literature describing mobile clinics dedicated to mental health.

Studies in Haiti have demonstrated high rates of depressive and anxious symptomatology as well as suicidal ideation among people living in rural areas [18–20]. One study found that 41% of patients presenting to a rural health clinic in 2010 complained of problems that may have been attributable to a mental disorder, but clinicians did not explore the psychosocial dimensions of the patients’ presenting problems in part due to insufficient access to mental health treatment [21]. Recognizing how few mental healthcare professionals worked in Haiti, several organizations implemented task-shifting mental healthcare initiatives using a range of models focusing on lay health workers in community-based settings [22–24], teachers in school-based settings [25–27], and primary care clinicians [28]. Starting in 2010, following a devastating earthquake, Zanmi Lasante (ZL), the Haitian affiliate of the international healthcare organization Partners In Health, developed a multi-tiered mental health program that included non-specialist mental health clinicians, community health workers (CHWs), and linkage to specialist mental healthcare located at 11 ZL-operated hospitals and primary care clinics [29–32]. In November 2011, patients with MNS disorders from one remote catchment area were referred to the team by a CHW, and many were subsequently lost to follow-up. When the team traveled to the area to follow up with these patients, it became clear that a community-based strategy was required because of the area’s remoteness and lack of alternative services. Our objective here is to describe the resulting innovative task-shifting approach to mental healthcare delivery in rural Haiti, a mental health mobile clinic, and its outcomes over the first two years alongside recommendations for implementation of similar approaches in other resource-limited settings.

## Methods

### Setting

The mobile clinic took place in the market town of Kas and mainly served the communities of Lahoye (pop. 15,417) and Tierra Muscady (pop. 13,318) in Haiti’s Central Plateau, northeast of Port-au-Prince [33]. The area is remote. It is not on a power grid and has no public works such as sanitation. Roads are unpaved and can easily become impassable during the rainy season, particularly for routes requiring crossing rivers.

Prior to the mobile clinic, people living in Kas had no access to mental healthcare. In 2011, a community-based epidemiological study in the area documented a high degree of mental health needs, including depressive and anxiety symptoms and suicidal ideation [19]. At the same time, a brief mental health intervention by another NGO trained several community health workers to identify and provide support for mental health problems, as well as establishing a referral system to ZL [19,24]. The nearest mental health services were in the village of Cange, 28km away from the Kas’ market town. For people living in Kas, where transportation consisted largely of walking or riding horses, the trip to Cange often took over four hours. By motorcycle taxi, often prohibitive in cost, the journey to the health center took at least one hour.

### Clinic overview

The mobile clinic was organized as a free daylong event and occurred every one to two months. A small church hosted the clinic. Its pews were arranged into clinician interview areas, set apart from each other to maintain patient privacy. It was typically staffed by two or three psychologists with bachelors degree-level training, two physicians (sometimes including a trained psychiatrist), one social worker, a non-clinician “pharmacist” to dispense medications prescribed by the physicians, and two CHWs.

The clinic was administered by the ZL Mental Health Team and funded locally by ZL.

CHWs were employed by ZL and another international nongovernmental healthcare organization working in the area. Initially, there was no additional compensation for their work in mental health or at the mobile clinic except for reimbursement for transportation to the clinic and lunch provided to the entire mobile clinic team. Later on, the ZL Mental Health Team instituted a small incentive payment for CHWs for incorporating mental health work into their other duties.

Several important services were *not* available at the clinic. Laboratory tests, radiography, and electroencephalography (EEG), if indicated, required a referral. Patients were expected to bring laboratory results to their next mobile clinic appointment or review findings with CHWs, who could then coordinate patient care with physicians for any medical concerns. Specialists (a psychiatrist or neurologist) were occasionally on site.

### Task-shifting

Task-shifting decisions at the mobile clinic, including mechanisms for training and supervision, were based on the platform in place at ZL (Table 1) [31]. A considerable amount of clinical activity at the mobile clinic occurred without direct specialist involvement (Table 1). Specialty tasks were managed by generalist physicians, bachelors-level psychologists, and CHWs. Task shifting occurred at all levels and involved medication prescribing, provision of psychosocial treatments, and screening for MNS disorders. For example, the psychiatrist treated neurological disorders through the use of a combination of treatment guidelines (such as mhGAP) and email consultation with neurology specialists. The generalist physicians learned psychiatry and neurology evaluations, reporting, and prescribing through apprenticeship with resident specialists, the use of guidelines, and ongoing supervision. Psychologists and social workers, who had bachelors-level training, were trained and supported to perform tasks typical of masters- or doctorate-level clinicians. They conducted evaluations and follow-ups, provided patient and family psychoeducation, and delivered a manualized psychotherapeutic intervention for patients with depression that was based on interpersonal therapy (IPT) and adapted to the Haitian context [34]. After a locally-developed screening tool (the Zanmi Lasante Depression Symptom Inventory (ZLDSI)) was introduced at the mobile clinic by psychologists in October 2012, the CHWs screened patients for depression symptoms [35].

**Table 1:** Level of education, relative investment of time on the project, and training, supervision, and distribution of responsibilities by provider and staff at the mobile clinic.

Provider/staff type	Level of education	Time on project^a^	Provider/staff responsibilities
Community Health Workers (CHWs)	High school	+++	Training: CHW curriculum (including modules for recognizing common mental disorders (i.e., those listed in mhGAP); psychoeducation for patients, family, and communities; psychological support / modified interpersonal therapy (IPT); referral process, including recognition of urgent matters; side effects of medication.) Supervision: Supervised by the CHW Supervisor who reviewed cases with CHWs, reviewed depression screening forms, asked patients to recount instructions provided by CHWs to assure fidelity to best practices, and co-facilitated community psychoeducation events Preparation: Community psychoeducation; Clinic planning at the community level (Working with Active case finding; Reminders Clinic day: Depression Screening; Registering patients Follow-up: Home visit to deliver medication, provide psychological support, to check patient progress to monitor side effects.
Non-clinician pharmacist / mental health team assistant or coordinator	Varied^b^	+	Training: On-site training including familiarization with the limited formulary; dispensing practices and record keeping; and reviewing dosing instructions with patients. Supervision: Supervised by generalist physicians who also reviewed reconciliation of prescriptions and medication. Preparation: Gather necessary tools and forms Clinic day: Review medication instructions with patient and dispense medications at mobile clinic pharmacy table Follow-up: Record keeping and data entry
Social workers	College	++	Training: Training and apprenticeship by specialist and nonspecialist physicians on evaluating and treating mental and neurological disorders; IPT training Supervision: Case review and supervision by the Director of Mental Health at ZL; Case review with nonspecialist physicians and specialist psychologists Clinic day: Mental health evaluation (in particular patients with mild mental health problems but complex social issues); Psychotherapy (IPT-based); Care planning with patients, families, and CHWs; Psychoeducation and stigma reduction counseling
Psychologists	College	++	Training: Training and apprenticeship by specialist and nonspecialist physicians on evaluating and treating mental and neurological disorders; IPT training Supervision: Case review and supervision by the Director of Mental Health at ZL; Case review with nonspecialist physicians and specialist psychologists Preparation: Consult with CHWs Clinic day: Mental health evaluation; Psychotherapy (IPT-based); Care planning with patients, families, and CHWs; Psychoeducation and stigma reduction counseling Follow-up: Supervise CHWs
Mobile Clinic / CHW Supervisor (senior psychologist)	College	+++	Supervision: Supervised by the Director of Mental Health at ZL Preparation: Clinic planning at the health system level (coordinating with the lead CHW; scheduling clinic; creating treatment team; organizing travel and logistics); Co-create best practices in CHW and psychological care Clinic day: Assign provider responsibilities; Perform psychologist duties Follow-up: Partner with CHWs to follow-up with critical patients; Supervise CHW activities
Non-specialist physician	Medical school	++	Training: Training on mental disorders based on mhGAP, apprenticeship from psychiatrist and neurologist when on-site Supervision: Supervision by the Director of Mental Health and by psychiatry or neurology consultants; Case review / continued education with specialists on-site or off-site using phone or internet-based telecommunications tools Preparation: Co-create best practices in prescribing; Coordinate formulary; Finalize patient’s list for the clinic day Clinic day: Triage; Medical evaluation; Prescribing Follow-up: Pharmacy reconciliation; On-call support for clinical emergencies
Psychiatrist and/or neurologist	Medical school and residency	+	Training: Usually some formal global health coursework, in addition to residency training; familiarization with mhGAP. Supervision: Bidirectional learning with local teams; Consultation with other specialists on challenging cases Preparation: Co-create best practices in clinical care Clinic day: Patient evaluation and consultation (some clinic days only) Follow-up: Supervision of non-specialist physicians and/or psychologists (in clinic or by teleconferencing);

a. Providers and staff spent time on the project both at the mobile clinic and in between mobile clinic days as follows

+ Circumscribed patient care or care coordination activity on the mobile clinic day and limited responsibility for patient care or clinic coordination between mobile clinic days

++ Full patient care or care coordination activity on the mobile clinic day and limited responsibility for patient care or clinic coordination between mobile clinic days

+++ Full patient care or care coordination activity on the mobile clinic day and full responsibility for patient care or clinic coordination between mobile clinic days

b. This position was filled by multiple persons including a CHW, an assistant to the ZL Mental Health program, a ZL laboratory technician, and the Partners In Health Mental Health Coordinator. The level of education varied from high school degree to a masters degree in public health. There was considerable flexibility in who could perform these non-clinical tasks.

If the psychologist or social worker diagnosed a condition for which medication treatment was indicated or an option, identified a patient who needed to be evaluated for comorbid medical illness or neurological problems, or needed a second opinion, the patient was seen by a generalist physician. Clinical staff collaborated with CHWs to assure that patients had appropriate surveillance and follow-up. Established processes of supervision and the many different opportunities to collaborate in the care of individual patients assured that the quality of care provided by nonspecialist providers was equivalent to specialist providers.

Mobile clinic staff became more proficient in their roles as they gained more experience. Generalist physicians attained competency in managing common MNS disorders through training (including apprenticeship to an in-country visiting psychiatrist), written resources (such as the Mental Health Gap Action Programme, mhGAP) [36], and in-person or tele-supervision. Specialists (psychiatrists and neurologists) supervised physicians. Challenging cases were presented, with advisement provided on history-taking, performing the physical exam, creating a differential diagnosis, and establishing a treatment plan. Psychologists received similar training with a focus on history-taking, diagnosis, understanding treatment options, and providing counseling and therapy. The ZL Mental Health Director (a masters-level psychologist) provided primary supervision that was supplemented by visiting psychiatrists and US-based, PhD-level psychologists. The majority of these task-shifting and supervisory relationships were established in advance of the mobile clinic as part of the broader ZL mental health model (Table 1) [30,31].

CHWs also required training to carry out their clinical role. With training and supervision, they were better able to actively identify cases of common mental disorders. CHWs worked closely with the CHW Supervisor on caring for persons with MNS disorders, including brief IPT, psychoeducation, and depression screening.

### Data sources and analytic strategy

There were three main sources of clinical data for the current analysis: medical charting of clinical encounters by providers, patient registries completed by CHWs, and pharmacy lists of dispensed medications. There were several instances of lost clinical documentation, including misplaced paper charts, patient registries, and pharmacy lists. This problem was addressed by creating an Excel-based electronic record on a password-protected computer updated on an ongoing basis during and after the clinic. For this analysis, clinical data were supplemented by a quality-improvement questionnaire and an interview with the lead clinic CHW.

To determine how well the mobile clinic met community mental healthcare needs, we conducted several analyses on the clinic’s first two years of data. Our objectives were (1) to understand access by examining why patients went to the mobile clinic, from where, and where they had previously sought care; and (2) to examine care provision, including services provided, retention of patients, and evolution of symptoms and clinical condition of patients diagnosed with a mental disorder.

Electronic patient records were analyzed in Excel and SAS 9.2. Records for all patients who visited the mental health mobile clinic between January 2012 and November 2013 were analyzed. Descriptive statistics were used to examine clinic attendance patterns by new and follow-up patients, patient demographics, distance and time to clinic, diagnoses, follow-up rates, interventions, referrals, and evolution of patient condition. The time required for the patient to travel between their home and the clinic site was estimated by the lead CHW based on patient addresses.

The quality improvement questionnaire was developed to understand potential gaps in provision of care. Questions assessed how patients heard about the mobile clinic, who referred them, where else they had sought care for the same problem and how far they traveled to reach the clinic. The questionnaire was completed by all patients presenting to the August 2013 clinic (N = 51). The CHW who registered patients completed questionnaires verbally and recorded responses. Descriptive statistics were used to analyze questionnaire results.

The CHW Supervisor conducted an in-depth interview with the lead CHW. Open-ended interview questions explored the process of patient recruitment and follow-up, examples of positive and negative outcomes among patients, challenges and community perceptions regarding the mobile clinic and areas for improving care provision and follow-up. The interview was audio-recorded and transcribed in Kreyòl. Thematic analysis was used to guide transcript review, identify themes, and select illustrative examples related to those themes [37].

### Ethical review

The ZL Ethics Committee approved all research activities. This research was considered exempt by the Institutional Review Board of the Harvard School of Public Health.

## Results

### Patient characteristics and clinic attendance

**Fig 1:**
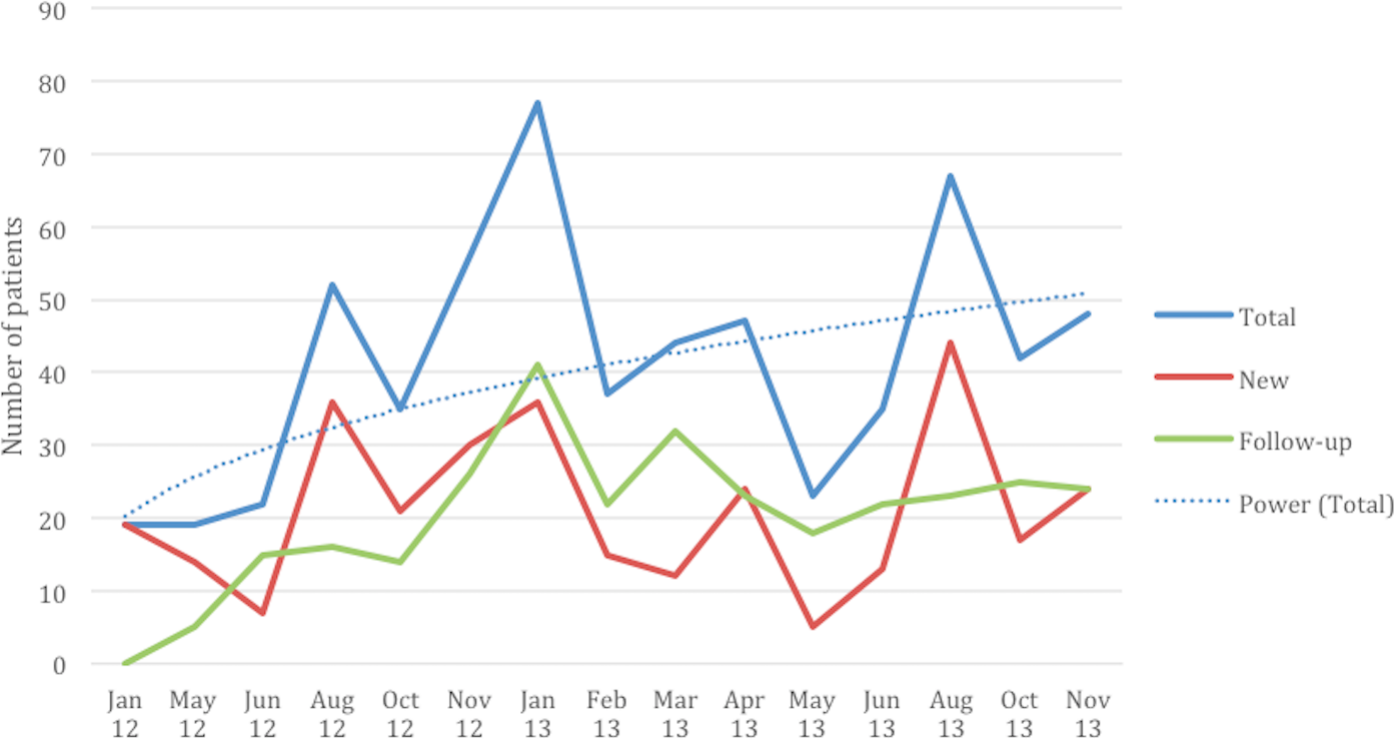
Number of mobile clinic attendees over time by follow-up status. Note: Patients without a documented diagnosis are included in Total category but neither of the other categories. There are various types of missing data that likely account for some fluctuation in attendance: A. Missing patient registration documentation: June 2012. B. Missing pharmacy disbursement information: August 2012, January 2013, February 2013, May 2013. C. Missing both types of information: January 2012, May 2012.

The clinic was well attended. Overall, we documented 318 unique patients between January 2012 and November 2013. Approximately two-thirds were female (64%), with a mean age of 37. Forty percent of patients lived within a half-hour walk of the clinic, while another 44% lived within an hour walk. Clinic records demonstrated that attendance fluctuated over the two-year period, ranging from a minimum of 19 to a maximum 77 documented clinic attendees at a single clinic (Fig 1).

Notwithstanding problems with documentation, some patterns emerged in clinic attendance data. Average clinic attendance showed an upward trend but this was not a consistent increase. There was some suggestion of seasonality to clinic visits, with decreased attendance during the rainy season (April-June). Variation in clinic volume mirrored the number of new patient visits, while follow-up patient visits did not increase. This may be due to multiple factors, including that some patients received a one-time consultation and others only needed to return every two or three months. Patient loss to follow-up also occurred. Some patients did not return to either the mobile clinic or a permanent clinic when referred, and at least one patient died (see Box 1).

**Box 1. A description of a patient raised by our CHW in the narrative interview when discussing some of the challenges of the mobile clinic**

In an early clinic, a young man with epilepsy and developmental delay arrived with myoclonus and mental status changes after experiencing several seizures the previous night. His mother reported that he had not returned to his prior level of functioning since the seizures started. Concerned for status epilepticus, the mobile team administered buccal lorazepam (in the absence of intravenous medications) [38], which quickly returned him to baseline. Psychologists provided psychoeducation about seizure precautions (including supervision when in the water or bathing) and the importance of adherence to medication. The patient was successfully treated with valproic acid had returned to the mobile clinic for follow-up until the rainy season, when he and his mother were unable to return because the rising river made the roads impassable. Before his CHW could bring him medication refills, the patient drowned after having a seizure while retrieving water. Unsupervised at the time, it suggested that seizure precautions were not followed. This may be a function of health literacy. It also reflects some of the realities of life in Haiti (bathing in rivers; open fires in homes; swollen rivers) that make it difficult for patients to observe seizure precautions or adhere to treatment faithfully. It also inspired the mobile clinic to reflect on how it might do things differently, such as providing extra medication to patients who may not be able to return easily to the clinic because of infrastructure problems. It also served as a reminder of the significant morbidity and mortality related to MNS disorders.

Patients were recruited through a mix of passive (word of mouth/self-referral) and active (CHW) strategies for case finding. Among those patients who completed the quality-improvement questionnaire (N = 51), 55% reported being referred to the clinic by a friend, family member, or pastor, and 43% reported being referred by a CHW. Approximately half (47%) reported having never accessed care previously for their problem. Patients reported previously seeking consultation for their problem from a medical doctor (59%), *hougan* (Vodou priest; 19%), or both (19%). One patient reported having consulted a doctor, CHW, and church.

### Patient diagnoses

The mobile clinic targeted persons with a wide range of MNS disorders. Of the unduplicated patients for whom diagnostic information was recorded (N = 235), 81% were diagnosed with a MNS disorder. Including follow-ups, the vast majority of clinic visits (90.2%) were utilized by patients with an MNS disorder. The most common MNS diagnosis were depressive disorders, accounting for one-third (33%) of patients, followed by epilepsy/seizure disorders (16%), and migraine/headache (12%) (Table 2). All other MNS disorders were diagnosed in fewer than 10% of patients. Approximately one-third of patients were diagnosed as having a non-MNS medical disorder such as anemia, hypertension, or gastrointestinal problems. Often comorbid with MNS disorders, general medical disorders were the second most common category of diagnosis (31%).

**Table 2:** Diagnostic categories of patients and follow-up visits by diagnostic category, for patients with recorded diagnosis (N = 235).

WHO diagnostic category	# Patients with diagnosis	% Patients with diagnosis	% Patients with diagnosis with any follow-up visits
Depressive disorder	78	33.2%	35.9%
Other medical condition	72	30.6%	27.8%
Epilepsy/Seizure disorder	37	15.7%	64.9%
Migraine/headache	28	11.9%	28.6%
Developmental/Behavioral disorder	18	7.7%	27.8%
Anxiety/Trauma/Stress	18	7.7%	38.5%
Movement disorder	11	4.7%	72.7%
Psychosis	12	5.1%	50.0%
Bipolar disorder	8	3.4%	75.0%
Other neurological	5	2.1%	40.0%
Dementia	2	0.9%	50.0%
Grief	2	0.9%	28.6%
Other psychiatric	2	0.9%	0.0%
Any diagnosis	235		34.9%
Any MNS diagnosis	192		40.1%

### Retention

We anticipated excellent retention in care by conducting the clinic in the community, but retention rates varied by diagnosis and treatment rendered. The highest follow-up rates were seen among those with diagnoses of bipolar disorder (75%), movement disorder (73%), and epilepsy/seizure disorder (65%) (Table 2). Approximately one-third of patients with depressive and anxiety disorders attended at least one follow-up visit. We did not find a significant difference in depression symptom severity between those with and without documented follow-up visits (independent samples t-test: t = -0.13; df = 103; p = 0.9).

### Care provision

Patients benefited from a wide array of mobile clinic services. Overall, 41% of patients received medication, 26% counseling, and 21% psychoeducation. The most common MNS-specific medications prescribed were carbamazepine, fluoxetine, and amitriptyline (20%, 18%, and 12% of MNS prescriptions, respectively). Approximately 40% of medications prescribed were provided for symptomatic relief of MNS disorders (analgesics for headache), folic acid for women of childbearing age receiving anti-epileptic drugs, or to treat non-MNS conditions (antibiotics for infection).

About half of all patients were referred for additional evaluation or tests. Twenty percent of patients were referred to a health center, including 10% for labs or studies, 7% referred to a specialist, and 6% referred for more frequent mental healthcare at the nearest health center. Of note, it is likely that this underestimates patient referrals to a psychologist as there was a lack of a standardized process to document referrals.

### Treatment outcomes

**Fig 2:**
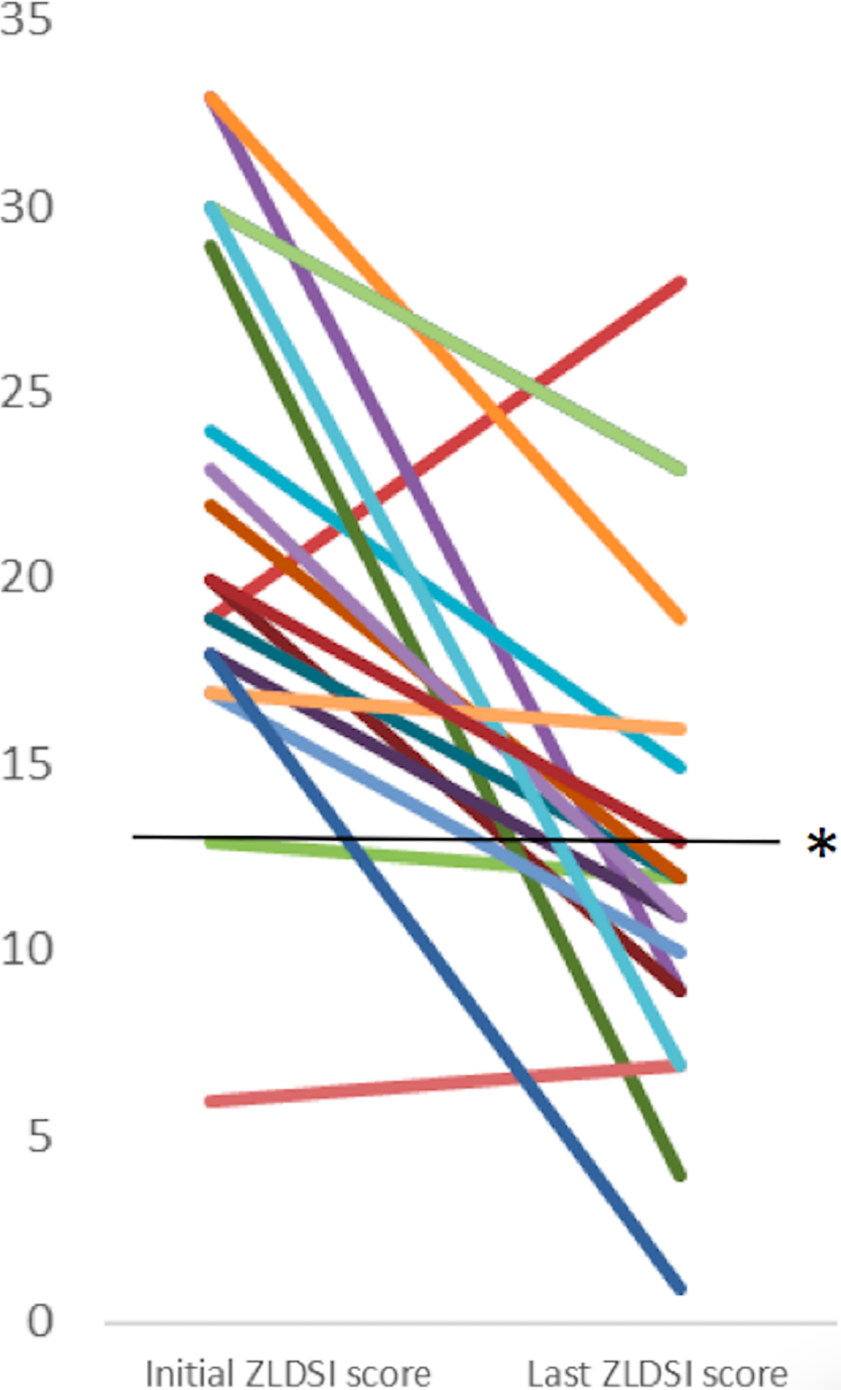
Initial and most recent follow-up scores on the Zanmi Lasante Depression Screening Inventory (ZLDSI) for patients with a current or past diagnosis of depression or bipolar disorder (N = 19). Colored lines represent depression severity scores of individual patients. The black line (*) represents the ZLDSI cut-off for mild depression (i.e., a score of 13).

Among patients with a documented score on the ZLDSI (N = 91), mean score was 17, which represents moderate depression. Among those with at least two documented scores on the ZLDSI (N = 22), mean change in score was a 9-point decrease (SD: 8.5; p < .001). Focusing on only those diagnosed with depression or bipolar disorder (N = 19), ZLDSI score changes dropped an average of 9.4 points (SD: 8.7; p < .001; see Fig 2).

### Barriers to community-based mental healthcare

In the interview, the lead CHW suggested that the mobile clinic addresses several challenges to the provision of mental healthcare in settings such as rural Haiti, namely: stigma, resource scarcity, and lack of availability of formal mental healthcare. He described clinical suffering and stigma prior to the mobile clinic:

“People used to suffer, they didn’t know what they had. There were people who would kill themselves, some would go to the Vodou priest, to the pastor; there are those who became worse until they died. They would stigmatize people, like those who have epilepsy. Myself, when I didn’t yet have information, hadn’t been trained, when they would fall [during a seizure], I was afraid to touch them. Now, I’m not afraid.”

He reported that clinic activities and patient improvement reduced stigma, which enabled patients to function better in communities. He described a patient from the Dominican Republic with an untreated MNS disorder who used to be placed in a wheelbarrow out of fear of making contact with him. Now, following treatment and stigma reduction activities, many community members accept him, and they no longer teased him and others like him.

## Discussion

Despite many challenges, this initiative demonstrated the feasibility and promise of a mobile clinic approach to the delivery of community mental healthcare in resource-limited settings. Bringing the clinic into the community shortened travel times for patients and their families and reduced the cost of obtaining mental healthcare. Similar to other initiatives to deliver psychiatric care at the clinic level [38], a diverse set of common MNS disorders were treated. Improvements in depression symptom severity were observed. We found suggestions that treating patients in a community-based setting was associated with a reduction of stigma.

This intervention overcame many of the barriers that are faced in providing mental healthcare in LMICs. First, many received mental healthcare for the first time at the mobile clinic. A direct comparison of services rendered at the fixed clinics and the mobile clinic is not possible, but the mobile clinic had certain advantages over fixed clinics: 1) the medical clinic in this community did not provide mental health care; and 2) the mobile clinic was more accessible, run by the same team, and had more active CHW participation than the nearest fixed clinic that did offer mental healthcare. Second, our clinic addressed one of the major challenges facing task-shifting interventions by providing a forum for ongoing, direct supervision [39].

Task-shifting and other resource-conscientious approaches hold great promise for expanding access to healthcare and were key to the mobile clinic’s success [40]. Transfer of clinical responsibilities to a Mobile Clinic Coordinator and to CHWs freed up human resources for other clinical projects and extended mental healthcare into a rural and remote community in Haiti. In envisioning improvement and scale-up, it is increasingly evident that CHWs played a critical role. This program benefited from having actively invested CHWs. This form of mental healthcare relies on the availability of highly motivated people [24]. We also faced similar problems as in other programs that rely heavily on CHWs, such as developing mechanisms to compensate CHWs to perform their vital role [41].

Despite its many successes, rates of follow-up were lower than expected, especially for depressive and anxiety disorders. Many factors could account for this observation. Some patients did not need follow-up and/or recovered. Also, the clinic did not provide financial support, which can be a deterrent to follow-up. Finally, it might be an artifact of documentation. For example, we could not document whether patients received follow-up psychotherapy at a permanent clinic rather than a subsequent mobile clinic. Examining how clinical, sociodemographic, and other factors influence follow-up rates and exploring ways to improve retention will be important moving forward.

### Limitations

This analysis has some limitations. The mobile clinic, like the broader mental healthcare initiative at ZL, was a dynamic process. Many processes evolved with time, the most significant of which were the roll-out of the ZLDSI. Because of the small number of patients who were administered the ZLDSI at two timepoints and the lack of other contextualizing data including a control group, it is difficult to make a strong statement regarding the relationship between treatment at the mobile clinic and the significant reductions in depression symptomatology that were observed. Improvements in record keeping were catalyzed by efforts to better describe clinic processes and outcomes. In early clinics, processes for documentation were not standardized. The short time-frame available for clinical encounters meant patient encounter forms were not always completed fully. Staff needed to develop a plan to maintain, organize, and transport hard copy patient records. Some patient information was undoubtedly lost or not effectively integrated into a patient’s record. Collectively, these challenges result in incomplete health records, which reduced providers’ ability to deliver effective care based on a comprehensive understanding of patients’ needs. Although it is unlikely that lost data would introduce bias into the clinic dataset, we may be underestimating clinic follow-up and other important outcomes.

This analysis has other limitations. Because the CHW Supervisor conducted the qualitative interview with the lead CHW, it is possible that that interview was influenced by social desirability bias limiting the discussion of mobile clinic flaws. Because the quality improvement survey was administered only on one clinic day, it is possible that the patients who attended the clinic that day were not representative of the clinic patients as a whole.

### Future directions

There is increasing recognition of the need for quality assurance practices in global mental health interventions and clinical trials of health service delivery. The use of formal, systematic, and/or quantifiable evaluation systems to document competencies of health providers and fidelity to best practices are recommended [42], and these would be useful at the mobile clinic as well. In addition, the value of a mobile mental health clinic could be tested empirically if efforts to expand the mental health clinic to other rural areas were to occur in the context of a community-based randomized trial.

Future innovations will involve strengthening processes for documentation, follow-up, and retention. Since the mobile clinic was initiated, ZL has undertaken a major initiative to improve data collection on patient care and integrate mental healthcare into the organization’s broader data collection systems. Technology-based mobile health innovations could help to improve follow-up, communication and coordination of care, and medical record keeping [8]. A pilot program using such technology was also implemented by ZL to learn more about how to optimize such innovations. By improving data quality, these interventions may facilitate analysis of mobile clinic outcomes and cost, and demonstrate sustainability of the project and its value to the broader health system.

## Conclusions

Here we have described a mobile mental health clinic as a novel way to provide community-based mental healthcare in a resource-limited setting. In the context of a broader initiative to integrate mental healthcare at the level of the health center, the mobile clinic addressed the mental health treatment gap by (1) using task-shifting to train and supervise non-specialist healthcare workers to deliver evidence-based mental healthcare and (2) providing access to care for persons in rural and remote communities. The efforts were associated with improved health outcomes and a reduction in stigma related to MNS disorders.
